# The Antisolvent Precipitation of CuZnOx Mixed Oxide Materials Using a Choline Chloride-Urea Deep Eutectic Solvent

**DOI:** 10.3390/molecules29143357

**Published:** 2024-07-17

**Authors:** William T. Wallace, James S. Hayward, Amy R. Marsh, Jonathan K. Bartley

**Affiliations:** Cardiff Catalysis Institute, School of Chemistry, University of Cardiff, Cardiff CF10 3AT, UK; wtw.twf@btinternet.com (W.T.W.); haywardj@cf.ac.uk (J.S.H.); amy.r.marsh@durham.ac.uk (A.R.M.)

**Keywords:** metal oxides, deep eutectic solvent, choline chloride, copper zinc oxide

## Abstract

Metal oxides have applications in a variety of different fields, and new synthesis methods are needed to control their properties and improve their performance as functional materials. In this study, we investigated a low-cost antisolvent precipitation method using a choline chloride-urea deep eutectic solvent to precipitate CuZnOx materials using water as the antisolvent. Using this methodology, the metal oxide materials can be precipitated directly from the deep eutectic solvent without the need for a high-temperature calcination step that can lead to a reduction in defects and surface area, which are important properties in applications such as catalysis.

## 1. Introduction

Copper-zinc oxide (CuZnOx) materials have a variety of applications as thin film semiconductors [1,2], ferromagnetic materials [3,4], antibacterial coatings [5,6,7,8], materials for energy production [9,10], and catalysts, most commonly for methanol synthesis via the hydrogenation of carbon monoxide and carbon dioxide and low-temperature water–gas shift reaction [11,12,13,14], although they have also been shown to be effective for low-temperature transformations of platform molecules from biomass [15,16] and as photocatalysts [17,18].

To control the properties, several different methods have been investigated for the synthesis of CuZnOx materials with the aim of improving their performance for the particular application of interest. Traditionally, CuZnOx materials have been prepared via routes such as coprecipitation [19], hydrothermal synthesis [6], or solid-state reaction [3], although these methods often lead to low surface area, as non-homogeneous materials contain single oxides rather than a single mixed oxide phase. Subsequently, a number of new synthetic routes have been developed to try and improve and control the properties of the mixed oxides, including hydrolysis [7,10], sol–gel spin coating [1,2], pulsed laser deposition [4], magnetron sputtering [20], spray pyrolysis [1,21], and electrodeposition [22]. However, these routes can require specialised equipment and be difficult to scale up.

We have previously studied alternatives to coprecipitation for synthesising CuZnOx catalysts. In coprecipitation, metal nitrates are precipitated from aqueous solution using sodium carbonate or sodium hydroxide, which generates a large amount of waste aqueous nitrate solution and aqueous base. In an attempt to make the synthesis process more sustainable, we previously studied alternative antisolvent precipitation methodologies that produce less waste. Initially, we investigated metal salt solutions that were precipitated by adding supercritical CO_2_ as an antisolvent. The precipitate is collected, and the solvent/antisolvent mixture can easily be separated downstream by dropping the pressure to give gaseous CO_2_ and the solvent, which can both be recycled in a closed loop. This method produced a novel georgeite phase precursor that, on activation, gave a more active CuZnOx catalyst for both water–gas shift [11] and methanol synthesis [12] when compared to coprecipitation. However, despite the improved performance, recycling CO_2_ between the supercritical phase at 150 bar for the precipitation and the gas phase at 100 bar for the separation is energy-intensive and not economically viable.

We subsequently turned our attention to low-pressure methods with a triethylamine/water solvent system to produce CuZnOx methanol synthesis catalysts, with promising activity [23]. Triethylamine, i.e., N(CH_2_CH_3_)_3_, can be switched between a hydrophobic biphasic mixture with water by bubbling CO_2_ through the system at low pressure to give a single-phase ionic liquid, i.e., [HN(CH_2_CH_3_)_3_]^+^[HCO_3_]^−^, and cycled back to the biphasic system by bubbling N_2_ through the solvent to allow for recovery and recycling of the triethylamine and water.

**Figure 1:**
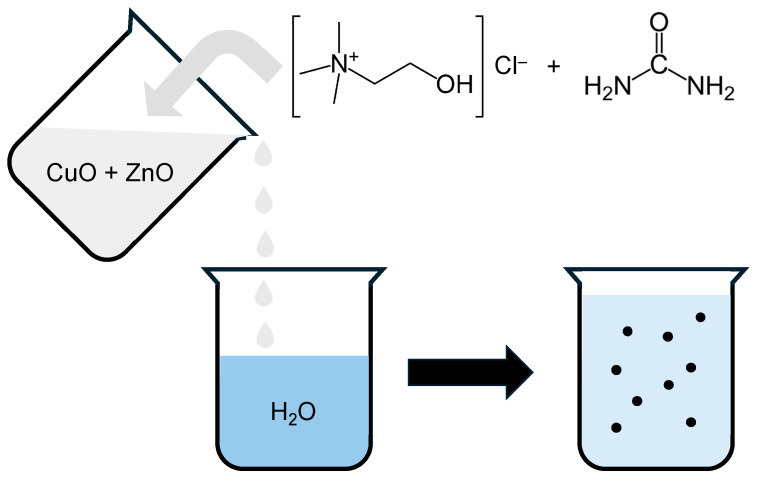
Schematic of the antisolvent precipitation process used in this study. Choline chloride as the hydrogen bond acceptor and urea as the hydrogen donor form a deep eutectic solvent (DES). Metal oxides are dissolved in the DES, and when the solution is added to the water antisolvent, the metal oxides are precipitated.

In the present study, we investigated a low-pressure antisolvent precipitation method using deep eutectic solvents as an alternative to the high-pressure supercritical CO_2_ process. Deep eutectic solvents (DES) are a group of ionic liquids based around quaternary ammonium salts, in particular choline chloride (Figure 1) [24,25,26,27,28].

The choline chloride is mixed with a hydrogen bond donor, which results in hydrogen bond interactions between the DES components leading to a significant decrease in melting point of the homogenous mixture compared to the individual components. DES are biodegradable, cheap, non-toxic, and do not react with water, making them a promising alternative to both traditional solvents and conventional ionic liquids. An interesting feature of DES is that a number of metal oxides can be dissolved by choline chloride-based DES. Particularly of interest to this study, ZnO can be dissolved in choline chloride-urea-based DES with a concentration of 1800–90,000 ppm depending on the temperature of the solution [27,28,29]. It was demonstrated by Dong et al. that zinc oxide could be precipitated out of the DES though an antisolvent method [30], primarily with water, and that different rates of addition of the antisolvent to the DES could change the morphology of the precipitated zinc oxide. Therefore, this methodology has the potential to synthesise metal oxide materials directly, without the need for high-temperature calcination to convert precursor salts into the metal oxide.

This study describes the investigation of different antisolvent methodologies for the synthesis of CuZnO materials using choline chloride-urea-based deep eutectic solvent systems using water as the antisolvent.

## 2. Results and Discussion

The synthesis of low loadings of copper (<10%)-doped zinc oxide using DES was previously investigated by Dong et al. [30], who dissolved zinc oxide in choline chloride-urea and copper nitrate in a water-ethylene glycol antisolvent. When the two solvents were mixed, a Cu^2+^-doped ZnO material was precipitated. We investigated a similar approach, using a solution of zinc oxide in choline chloride-urea solvent with an aqueous solution of copper acetate as the antisolvent. Using this methodology, a range of materials were investigated with different Cu:Zn ratios up to 2:1. During these experiments, the Cu:Zn ratio was controlled by dissolving different amounts of copper acetate in the same volume of the water antisolvent.

**Figure 2:**
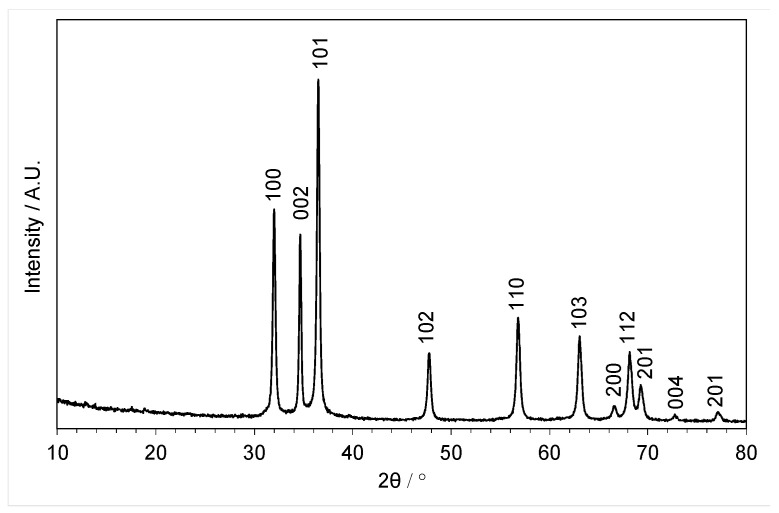
XRD pattern of ZnO (PDF 36-1451) (2800 ppm) precipitated from the choline chloride (7.0 g)-urea and (6.0 g) DES with 1500 mL of water as the antisolvent.

The XRD pattern of the material precipitated in the absence of copper confirms that zinc oxide can be directly obtained using this antisolvent approach (Figure 2), with the expected reflections at 32.0° (100), 34.6° (002), and 36.5° (101) along with 47.7° (102), 56.8° (110), and 63.1° (103) (JCPDS 36-1451). Previous studies on the precipitation of ZnO from choline chloride-based DES found that zinc carbonate can form as a result of a reaction of zinc oxide with urea during the antisolvent process [31], but no additional reflections to indicate any crystalline impurities were observed in this pattern.

**Figure 3:**
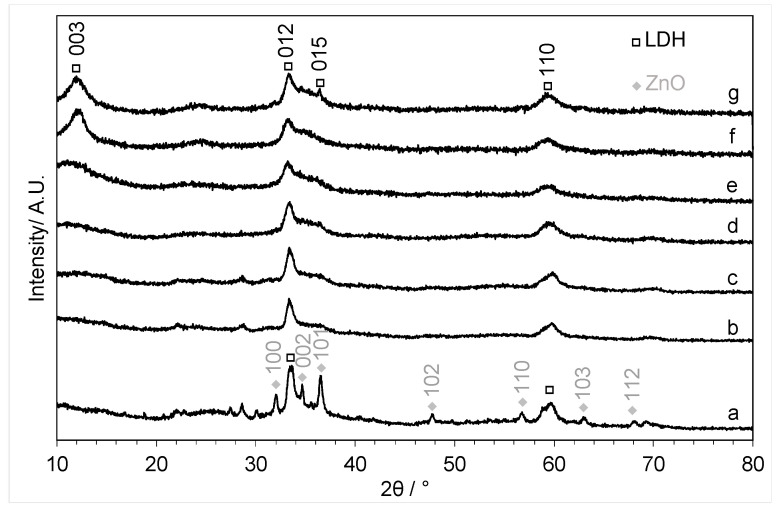
XRD patterns of the copper-zinc materials precipitated using the fast mixing methodology with Cu:Zn ratios of (a) 0.01:1, (b) 0.02:1, (c) 0.05:1, (d) 0.1:1, (e) 0.2:1, (f) 0.4:1, and (g) 1:1. Major peaks of the layered double hydroxide (LDH) (PDF 00-014-0191) and ZnO (PDF 36-1451) are shown.

The XRD patterns of the materials that were precipitated in the presence of copper acetate are shown in Figure 3. For the lowest loading of copper (Cu:Zn = 0.01:1), zinc oxide reflections were still visible. However, with increased copper loading, the zinc oxide reflections started to disappear, and the XRD shows a less crystalline material was formed that could be indexed to a (hydrotalcite-like) layered double-hydroxide (LDH) phase [32,33] (JCPDS card 00-014-0191), indicating the formation of a mixed-phase material. Previously, the formation of Co-Fe LDHs was reported during solvothermal synthesis with choline chloride-urea after the addition of water [34]. Our results demonstrate that elevated temperatures are not required for the formation of LDHs.

**Figure 4:**
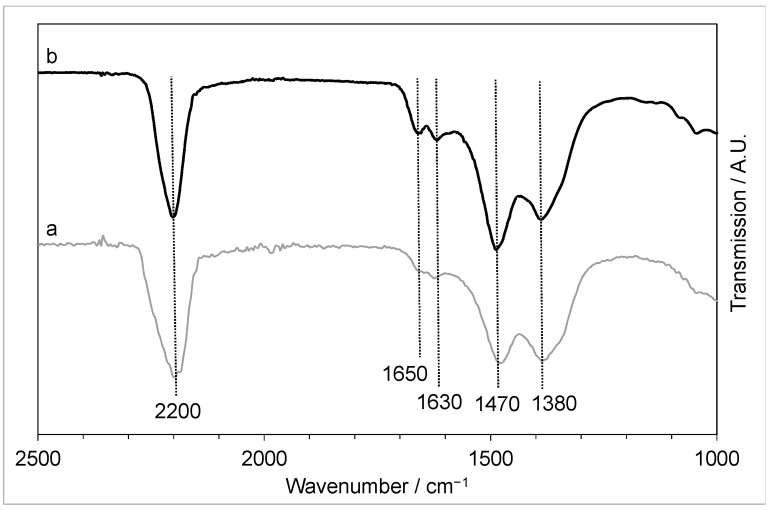
FTIR of the copper-zinc materials precipitated using the fast mixing methodology with Cu:Zn ratios of (a) 0.05:1 and (b) 0.4:1.

The FTIR of the materials (Figure 4) showed features that can be assigned to hydroxides, which would be expected from the LDH phase identified by XRD. However, there is also a strong band at 2200 cm^−1^ that suggests a possible urea–metal coordination bond, which is known to occur in metal salt-urea-based DES [35]. In addition, weaker vibrations at 1630 and 1650 cm^−1^ (C=O of urea) would confirm that some of the urea from the DES was precipitated and retained in the structure of the precipitate. It was shown that when dissolved in the DES, ZnO can form a complex with urea [28] and the metal-urea vibration seen in the FTIR suggests that urea is still bound to the zinc after precipitation. In addition, there are peaks, notably at 1470 and 1380 cm^−1^, that could be assigned to residual choline chloride.

**Figure 5:**
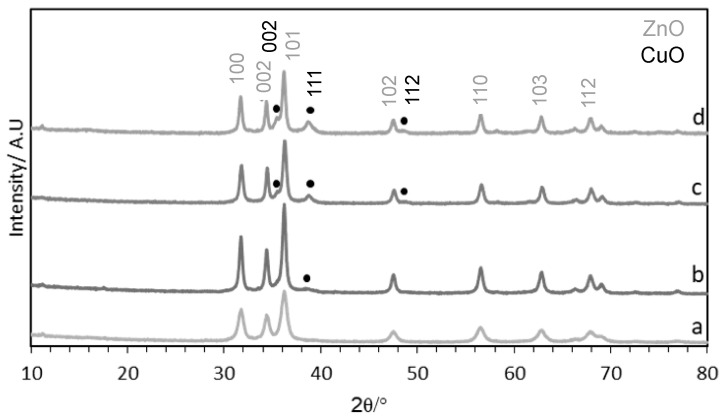
XRD of CuZnOx materials synthesised using the fast mixing methodology calcined at 350 °C with Cu:Zn ratios of (a) 0.05:1, (b) 0.1:1, (c) 0.2:1, and (d) 0.4:1. Major peaks of CuO (PDF 45-0937) and ZnO (PDF 36-1451) are shown.

The precipitated materials were then calcined at 350 °C for 3 h. The XRD patterns of the samples (Figure 5) confirmed the formation of CuO/ZnO after calcination and the CuO reflections (notably at 2θ = 38.6°) becoming more intense with increased copper loading.

**Figure 6:**
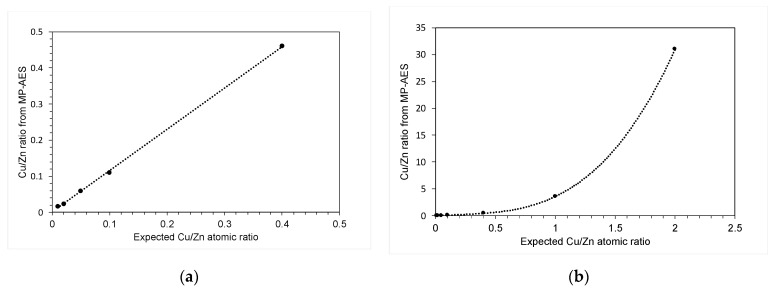
The expected Cu/Zn atomic ratio from the stoichiometry of the experiment plotted against the Cu/Zn ratio determined from microwave plasma atomic emission spectra: (**a**) Cu:Zn = 0.01:1 to 0.4:1; (**b**) Cu:Zn = 0.01:1 to 2:1.

However, MP-AES analysis on the calcined materials (Figure 6) showed that the target loading of copper was only achieved with low Cu:Zn ratios (≤0.4:1), and when the target ratio of Cu:Zn was increased, MP-AES showed a higher-than-expected copper loading, suggesting that more concentrated solutions of copper acetate prevent the zinc oxide from precipitating. At high Cu:Zn ratios, there are two orders of magnitude more copper acetate ions dissolved in the water. This can influence the properties of the solvent, including lowering the pH of the solution, allowing Zn-O bonds increasing the solubility in the water, and decreasing its ability to act as an antisolvent.

**Figure 7:**
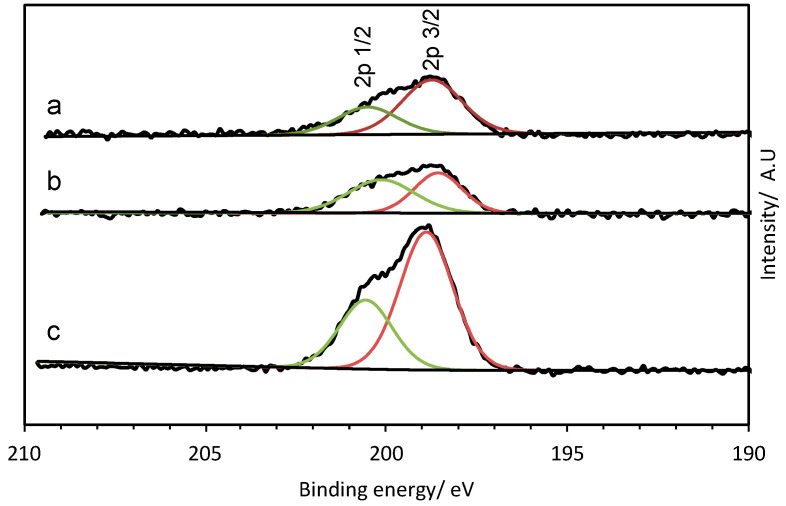
XPS of Cl 2p region of CuZnOx materials synthesised using the fast mixing methodology after calcination at 350 °C: (a) 0.4:1, (b) 0.2:1, and (c) 0.05:1. The green and red lines indicate the peak fitting of the Cl 2p 1/2 and Cl 2p 3/2 contributions respectively.

XPS analysis of the Cl 2p region of the calcined CuO/ZnO (0.05:1, 0.2:1, and 0.4:1) (Figure 7) showed that there was a significant amount of chlorine (4.5–12.5 at.%) left on the surface of the catalyst even after calcination. The spectra showed a doublet of peaks at 198.9 and 200.8 eV corresponding to the 2p 3/2 and 2p 1/2 binding energies, respectively [36], which indicates that the surface chloride is in the form of a metal chloride rather than residual organic chloride.

The effect of solvent/antisolvent mixing was investigated by modifying the methodology in an attempt to increase the rate of precipitation. In our previous studies investigating supercritical CO_2_ precipitation, we found that rapid mixing of the supercritical antisolvent and solvent led to a rapid increase in supersaturation of the solution. This led to a precipitation mechanism where nucleation dominated over crystal growth, leading to small, amorphous particles with a high surface area [11,12,37,38,39,40]. 

In this study, the initial experiments were carried out by pouring the DES solution into a large excess of the water antisolvent. This methodology is likely to lead to a fast precipitation, as the rate of diffusion between the two liquids will be fast. The methodology was then modified to inject a flow of the aqueous solution into the DES at a constant rate. This methodology has a much smaller ratio of water to DES, so there is less antisolvent present (particularly at the start of the reaction) to initiate nucleation. It was therefore expected that this methodology would lead to larger particles and a low-surface-area material.

**Figure 8:**
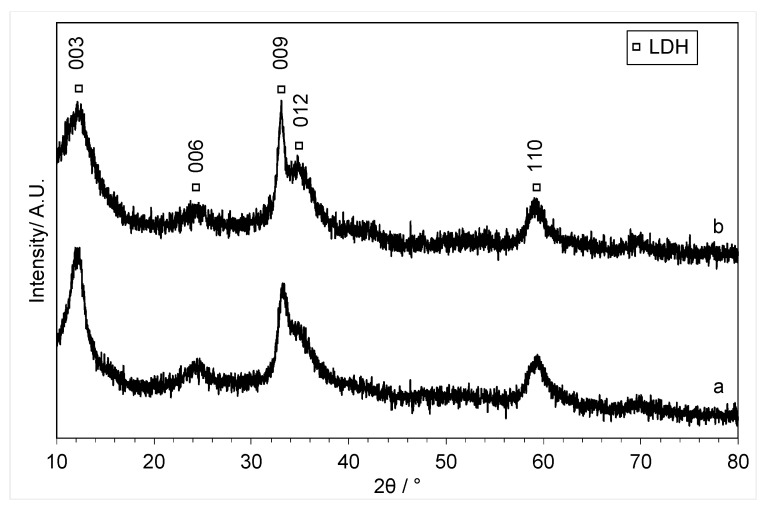
XRD of the 0.4:1 Cu:Zn material precipitated with (a) fast mixing and (b) slow mixing of the DES and antisolvent. Major peaks of Layered double hydroxide (LDH) (PDF 00-014-0191) are shown.

The XRD (Figure 8) of the precursor that was formed with a slow addition of the copper acetate water antisolvent showed the same pattern that could be indexed to the LDH-like phase, which is similar to the material precipitated by rapid addition, showing that the rate of addition did not alter the composition of the material (Figure 3).

**Figure 9:**
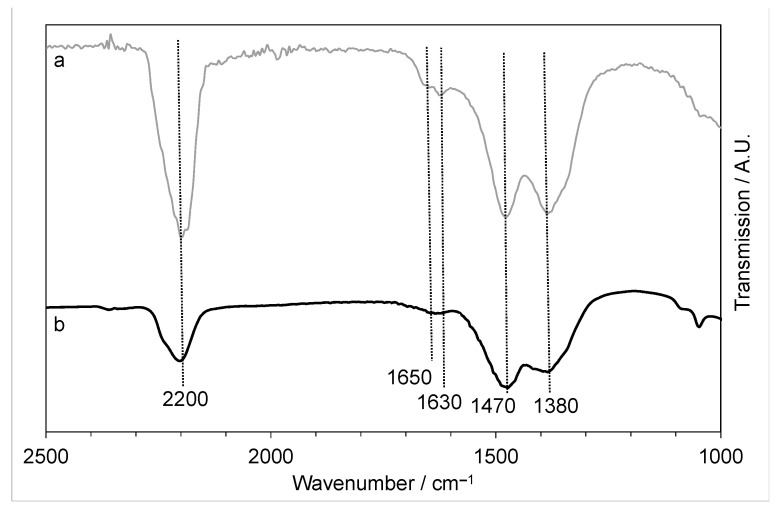
FTIR of the 0.4:1 Cu:Zn material precipitated with (a) fast mixing and (b) slow mixing of the DES and antisolvent.

FTIR (Figure 9) also confirmed that the same precursor was produced with the different rates of precipitation, both showing the hydroxyl peak (3380 cm^−1^), the metal-urea shift (2200 cm^−1^) [35], the CO shift from urea at 1630–1650 cm^−1^, and carboxyl shifts at 1470 and 1380 cm^−1^. This indicates that the slow-precipitated copper-zinc precursor will still have some DES retained in the sample, so it is likely that the final CuZnOx will still have large amounts of chlorine present.

While the XRD and FTIR showed little difference between the two rates of addition, BET analysis of the precursors reviled a rapid increase in the surface area from 59 m^2^ g^−1^ to 212 m^2^ g^−1^, demonstrating that the rate of addition had an impact on precursor morphology without altering the phase. However, on calcination, the surface area of both materials dropped to around 40 m^2^ g^−1^, possibly due to the exothermic decomposition of residual choline chloride in the sample causing sintering of the materials.

When these materials were tested as methanol synthesis catalysts, they were found to give very low CO conversion, which was attributed to a combination of the chloride poisoning and low copper surface area (<0.5 m^2^ g^−1^). To try and address the issues with residual chloride and non-stoichiometric precipitation at high Cu:Zn ratios, an alternative methodology was investigated where CuO and ZnO were both dissolved in the deep eutectic, and water added as the antisolvent to induce precipitation (Figure 1). The solubility of copper oxide has been previously investigated in different choline chloride-based DES [28,29]. With urea as the hydrogen donor, CuO was only slightly soluble at 50 °C (4.8 ppm), although this increased considerably at 70 °C (234 ppm). However, unlike ZnO, the metal–oxygen bond is broken, and ionic CuCl_2_ is often precipitated. The use of oxalic or malonic acid as the hydrogen bond donor can improve the solubility but forms complexes similar to sol–gel syntheses, which can result in metal oxalates or malonates being precipitated. Higher ratios of Cu:Zn were explored to investigate whether stoichiometric precipitations could be achieved using this methodology.

**Figure 10:**
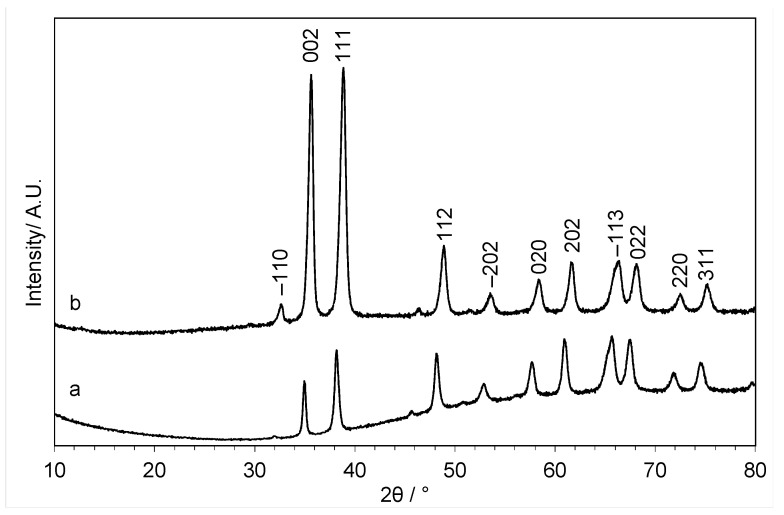
XRD patterns of CuZnOx materials precipitated from a solution of CuO (PDF 45-0937) and ZnO in choline chloride-urea DES with water as the antisolvent with (a) Cu:Zn = 1:1 and (b) Cu:Zn = 7:3. Major peaks of CuO (PDF 45-0937) are shown.

In the new methodology, CuO and ZnO were dissolved together in choline chloride-urea with Cu:Zn ratios of 1:1 and 7:3 and precipitated with water as the antisolvent. The XRD patterns of the materials obtained are shown in Figure 10. The materials produced using this methodology have a very different XRD pattern when compared to the LDH patterns obtained using the previous methodology (Figure 3 and Figure 8). Both patterns could be indexed to CuO, with reflections at 2θ = 35.7°, 38.9°, 49.2°, 58.5°, and 62.0° corresponding to the (002), (111), (112), (020), and (202) planes, respectively [41], which were seen previously for the calcined catalysts prepared using the previous methodology (Figure 5). This is a surprising result, as the change in methodology to dissolve both metal oxides in the choline chloride-urea DES not only improved the yield of the precipitation process but also allowed the reprecipitation of the dissolved oxides rather than CuCl_2_, as observed with the aqueous solution of copper. Although ZnO reflections were not observed, the CuO peaks are broad and could be covering the ZnO pattern. It is also possible that this methodology produced small ZnO particles that are below the detection limit of X-ray diffraction.

## 3. Materials and Methods

### 3.1. Choline Chloride-Urea Deep Eutectic Solvent Synthesis

In this work, choline chloride, i.e., (CH_3_)_3_NCH_2_CH_2_OH)Cl (98%, Sigma Aldrich, Glasgow, UK), was mixed with urea (CO(NH_2_)_2_, 99.5%, Sigma Aldrich) in a 1:2 molar ratio. The formation of deep eutectic solvents outlined here is well established in the literature [26,27,28,29,30]. First, 6.0 g (0.1 mol) of urea and 7.0 g (0.05 mol) of choline chloride were mixed together in a round-bottom flask. The mixture was heated to 82 °C, and after 2 h, a clear homogeneous liquid formed. 

### 3.2. Copper Zinc Oxide Synthesis Using Choline Chloride-Urea Deep Eutectic Solvent

Two different experimental procedures were investigated to precipitate the Cu-Zn materials from a choline chloride-urea deep eutectic solvent with water as the antisolvent.

#### 3.2.1. Method One

In the initial experiments, a solution of zinc oxide in choline chloride-urea solvent was mixed with an aqueous solution of copper acetate as the antisolvent. The rate of antisolvent addition was also investigated using this methodology.

Zinc oxide (ZnO, 99%, Sigma Aldrich) was dissolved in the choline chloride-urea DES (see Section 3.1) with a zinc concentration of 28,000 ppm at 82 °C and left for 2–3 days to dissolve to form a single-phase solution. 

##### Fast Mixing

The ZnO solution was poured into deionised water (1500 mL) containing copper acetate, with the concentration depending on the target loading. A blue precipitate formed, which was then filtered and washed with deionised water and ethanol and dried at 60 °C for 15 h. The precipitates were calcined in flowing air (10 mL min^−1^) at 350 °C for 3 h with a heating rate of 5 °C min^−1^. 

##### Slow Mixing

Copper acetate was dissolved in 100 mL of deionised water and injected into the ZnO containing the deep eutectic mixture at a rate of 1 mL min^−1^, followed by 600 mL of deionised water at a rate of 2 mL min^−1^. Using this methodology, a range of materials were investigated with different Cu:Zn ratios (from 0.01:1 to 2:1), with the Cu:Zn ratio controlled by dissolving different amounts of copper acetate in the same volume of the water antisolvent. The precipitate was then filtered and washed with deionised water and ethanol and dried at 60 °C for 15 h. The precipitates were calcined in flowing air (10 mL min^−1^) at 350 °C for 3 h with a heating rate of 5 °C min^−1^.

#### 3.2.2. Method Two

The second methodology involved dissolving both the zinc oxide and copper oxide in the deep eutectic mixture and then mixing with water as the antisolvent. 

The metal oxides were added to the DES (see Section 3.1) with a choline chloride: metal oxide ratio of 2:0.1. This mixture was then heated at 82 °C for 2/3 days until all the metal salt had dissolved. Once dissolved, the deep eutectic mixture was then added into 600 mL of the solvent (deionised water) and left under vigorous stirring for 1 h. The solution was then filtered under vacuum and washed with deionized water and ethanol before being dried in the oven.

### 3.3. Material Characterisation

#### 3.3.1. Powder X-ray Diffraction

XRD patterns were obtained using a PANalytical X’pert Pro diffractometer (Malvern Panalytical, Malvern, UK) equipped with a Cu Kα source at 40 kV and 40 mA. The samples were placed in an Al sample holder, and scans were taken from a 2θ angle of 5° to 80° at a scan rate of 2° min^−1^. PANalytical Highscore was used to match the XRD data to the crystal group and identify the sample phase using the International Centre for Diffraction Data (ICDD) database.

#### 3.3.2. FTIR

Infra-red spectra were recorded on a Vertex 70 (Bruker, Coventry, UK) equipped with an ATR cell and a mercury cadmium telluride (MCT) detector. A background of 16 scans was recorded prior to the sample scan. The sample was then placed in the ATR cell and pressed onto the crystal and exposed to the IR beam for 32 scans in absorbance mode with an aperture setting of 6 cm^−1^. 

#### 3.3.3. Microwave Plasma Atomic Emission Spectroscopy 

For MP-AES analysis, 50–75 mg of the sample was dissolved in 10% (volume) aqua regia (1:3 HCl:HNO_3_) in aqueous solution and left overnight to digest. The solution was then diluted so that the total concentration of metals in the mixture was approximately 10–30 ppm. Reference solutions of the metal from 1000 ppm stock solutions (dissolved in HNO_3_/water) were made to concentrations of 5 ppm, 10 ppm, 20 ppm, and 30 ppm to calibrate the MP-AES before analysing the solution. Each metal concentration was analysed using two wavelengths so that an average concentration could be determined. 

#### 3.3.4. X-ray Photoelectron Spectroscopy

The XPS analysis was carried out using a Thermo Fischer Scientific (Newport, UK) K-alpha+ spectrometer. Samples were analysed using an Al X-ray source (72 W) over an elliptical area of 400 μm × 800 μm. The samples were charge-neutralised using argon ions and electrons. For survey scans, the data were recorded with energy passes of 150 eV with a 1 eV step and for high-resolution scans with a pass of 40 eV (1 eV step). Data analysis was carried out using CasaXPS V2.3.23 [42]. 

#### 3.3.5. Surface Area Determination

The 5-point surface area analysis of the samples was performed on a Gemini 2360 (Micromeritics, Tewkesbury, UK). First, 100 mg of the sample was placed into a 9.6 mm × 155 mm tube and degassed under nitrogen on a FlowPrep 060 (Micromeritics, Tewkesbury, UK) at 110 °C for 1 h for metal oxides and 60 °C for 16 h for precursor materials. 

## 4. Conclusions

In this study, we demonstrated that CuZnOx materials can be produced using a simple, inexpensive, antisolvent methodology using a choline chloride-urea deep eutectic solvent and water as the antisolvent. The methodology that used a solution of ZnO in a choline chloride-urea and copper acetate in aqueous solution precipitated a layered double-hydroxide material that, on calcination, formed a mixture of CuO and ZnO. Altering the conditions so that the solvent and antisolvent were mixed more slowly resulted in a high-surface-area precursor, although the surface area was not retained in the final catalyst. Using this methodology, ZnO could be precipitated directly without the need to heat treat the catalyst to decompose a precursor material. 

When the precipitation process was modified so that both CuO and ZnO were dissolved in the choline chloride-urea deep eutectic and precipitated with water, the XRD of the precipitated material could be indexed to CuO (although it is likely that ZnO is also present), indicating that tuning the methodology can enable different metal oxides to be precipitated directly. Although this study was carried out at a relatively small scale in batch mode, there is a potential for scale up by investigating the precipitation process in flow. 

The high residual chlorine content in the materials from the choline chloride meant that they showed poor catalytic performance for CO hydrogenation, although alternative choline anions or solvent systems could allow for a chloride-free synthesis methodology. However, it is possible that the methodology could have potential for other applications. The ability to directly form metal oxides at low temperatures means a calcination step is not required, which could be beneficial to prevent sintering in applications where high-surface-area materials are needed. 

## Data Availability

The original data presented in the study are openly available free of charge through the Cardiff University Research Portal (https://doi.org/10.17035/d.2024.0327139688).
